# Ugandan Physician Attitudes towards a Potential, Local Trauma Fellowship Program

**DOI:** 10.21203/rs.3.rs-5688403/v1

**Published:** 2025-01-01

**Authors:** Alan Zambeli-Ljepović, Treasure Ibingira, Caroline Stephens, Rachel Koch, Marissa A. Boeck, Doruk Ozgediz, Martha Namugga

**Affiliations:** University of California San Francisco; Makerere University College of Health Sciences; University of California San Francisco; University of California San Francisco; University of California San Francisco; University of California San Francisco; Mulago National Referral Hospital

**Keywords:** Trauma, injury, surgery, training, Uganda, fellowship, low resource, low income, global surgery, survey

## Abstract

**Background:**

In low-income countries, clinicians trained through a context-specific trauma surgery fellowship program (TFP) can help reduce injury-related mortality to levels closer to those observed in higher-resource settings. Successful implementation, however, hinges on buy-in from local clinicians. We therefore assessed clinician support for a potential TFP in Uganda, considering perceived need, curricular recommendations, barriers, and motivating factors.

**Methods:**

After cognitive interviews with experts and questionnaire pilot testing, we cross-sectionally surveyed Ugandan consultants (general surgeons and procedural specialists involved in trauma care) and surgical residents at a tertiary, national referral hospital. Respondent percentages were calculated for multiple-choice answers, and we performed thematic analysis of free-text responses using a primarily inductive approach.

**Results:**

Among 46 faculty (from 13 specialties) and 42 resident respondents, 86% supported a Ugandan TFP. Respondents recommended incorporating emergency general surgery (66%), critical care (84%), and international rotations (76%) into the curriculum. Severe resource and structural deficiencies (82%) and concern about governmental support for post-training employment and compensation (66%) were leading perceived barriers to TFP implementation. Most faculty felt a TFP would improve patient outcomes (93%), overall trainee education (77%), and clinical efficiency (68%). Free-text responses were consistent with survey themes, indicating acute awareness of current trauma system inadequacies and conviction that a TFP would reduce injury-related mortality.

**Conclusions:**

Ugandan clinicians who care for injured patients view a TFP as crucial to improving injury-related outcomes, despite known barriers. TFP implementation should incorporate curricular recommendations from this survey and address widespread concerns about financial and infrastructural support from the national government and local institutions.

## Background

Worldwide, injuries claim over four million lives every year, exceeding the combined annual mortality from malaria, tuberculosis, and human immunodeficiency virus [1]. For every injury-related death, 10 additional injured people survive to live with permanent disability [1]. More than 90% of this global burden comes from low- and middle-income countries (LMICs), and often affects the youngest, most productive people within the population [2].

Uganda represents a microcosm of this global problem. Like other countries in its region and income level, Uganda faces the combined challenge of a high injury burden and a system that is ill-equipped to manage it [3, 4]. Despite road safety efforts by the Ugandan Police Force, infrastructural barriers and increasing motorcycle use have contributed to rising road traffic injuries and fatalities [5–7]. On the receiving end, Ugandan hospitals, from the district to the national level, are limited by deficits in supplies and trained personnel, lack of standardized trauma resuscitation processes, and inconsistent ownership of injured and emergently admitted patients [8–11]. Our research group focuses on surgical workforce strengthening, which is a critical factor in reducing injury-related mortality [12, 13], enables effective leverage of international partnerships [14], and is the aspect that surgeons at Mulago National Referral Hospital (MNRH), in Uganda’s capital of Kampala, feel is most within their control.

Specifically, we are focused on the development of a trauma fellowship (subspecialty) training program (TFP), which currently does not exist in Uganda nor in any of its 13 peer nations in the College of Surgeons of East, Central and Southern Africa (COSECSA) [15]. Similar programs have improved patient throughput and surgeon satisfaction in high income countries, and although data from LMICs are sparse, initial results for an orthopedic TFP in Haiti show the potential for similar benefits [16, 17]. Since the success and sustainability of such an intervention hinge on contextual relevance and support from local clinicians [18, 19], our group of Ugandan surgeons and United States-based trauma surgery specialists aimed to evaluate Ugandan clinicians’ perceptions of a potential locally run TFP, including perceived utility of such a program and the potential barriers and motivating factors pertaining to its establishment and operation. We hypothesized that most clinicians would support the development of a locally run TFP.

## Methods

We performed a cross-sectional, multiple-choice and free-response survey of Ugandan (1) physician specialists who have finished their training and routinely care for trauma patients (henceforth, “consultants”) and (2) surgical residents. We followed the Consensus-Based Checklist for Reporting of Survey Studies (CROSS) [20].

### Survey Design

Surveys were designed by our multinational research team of surgeons from different specialties, all of whom work at academic hospitals and regularly care for injured patients. While some questions applied to both consultants and residents, we designed separate surveys tailored to respondents’ specific experiences. For example, consultants were asked to rank the most important skills from their specialty, in which they believe a Uganda trauma surgeon should be proficient; residents were asked to indicate their interest in pursuing a trauma fellowship after residency. We refined the surveys through cognitive interviews with two Ugandan surgeons from different specialties (MN and TI), two U.S.-based surgeons (DO and MB, including one trauma consultant), and two U.S.-based surgical residents (CS and AZL). Finally, we pilot tested the surveys with Ugandan and U.S.-based consultants and surgical residents prior to dissemination.

The consultant survey comprised 20 questions; the resident survey had 14. All questions were multiple choice except the last, which was an optional free-text prompt soliciting additional comments about a potential TFP in Uganda.

### Study Population and Sampling Technique

We identified consultants through professional networks, including the Association of Surgeons of Uganda. To represent all potential faculty who would be teaching a trauma fellow, we included consultants from all 13 disciplines involved in trauma care at MNRH. We included all surgical residents training at MNRH, the largest surgical training hospital in the country and the one offering the largest exposure to trauma care. We excluded surveys that were less than 75% complete. We asked participants to provide their phone numbers, which were used for remuneration (see “Ethical Considerations”) and to detect multiple responses by a single participant.

To obtain representative results at a 95% confidence level and with a 5% margin of error, we aimed to obtain survey responses from 80% of our target population. For residents, given a target population of 55 surgical residents at MNRH, our target sample size was 44. For consultants, since we distributed surveys broadly though professional society networks, it was not possible to determine the size of the target population or a response rate. We therefore used quota sampling to ensure at least one consultant from each discipline had completed the survey.

### Ethical Considerations

We obtained ethical clearance from the MNRH Institutional Review Board. At the start of the survey, participants were given an information sheet explaining the purpose of the study, a brief description of a TFP, and the option to decline completing the survey. By proceeding with the survey, participants indicated their informed consent to participate in the study. Upon survey completion, each participant was given monetary participation to acknowledge their time and effort.

### Survey Administration

After obtaining ethical clearance and pilot testing, the senior author (MN) and an administrator in the MNRH Department of Surgery emailed electronic survey links (Qualtrics 2024, Provo, Utah, U.S.) to surgical residents and consultants. The survey links, accessible by phone or computer, were emailed twice weekly over six weeks (January to February 2024), until the following criteria were met: (1) the resident survey reached the sample size target (80%) and (2) at least one clinician from each specialty had responded.

### Data Analysis

Quantitative results from multiple-choice questions are reported as frequencies and percentages. For the single ranking question in the survey, we reported the top three ranked answers for each question; fewer if there were fewer than three choices provided. For the free-text responses, two authors (AZL and MN) performed a reflexive thematic analysis [21], acknowledging the positionality of the research team, which includes both Ugandan and U.S.-based clinicians, all of whom have worked, in varying clinical capacities, in Ugandan hospitals. The analysis was primarily inductive, using semantic coding initially. Subsequent passes focused on uncovering latent meaning in the responses and cross-checking their implications based on the study team’s own understanding of healthcare in Uganda.

## Results

### Respondent Characteristics

**Figure 1:**
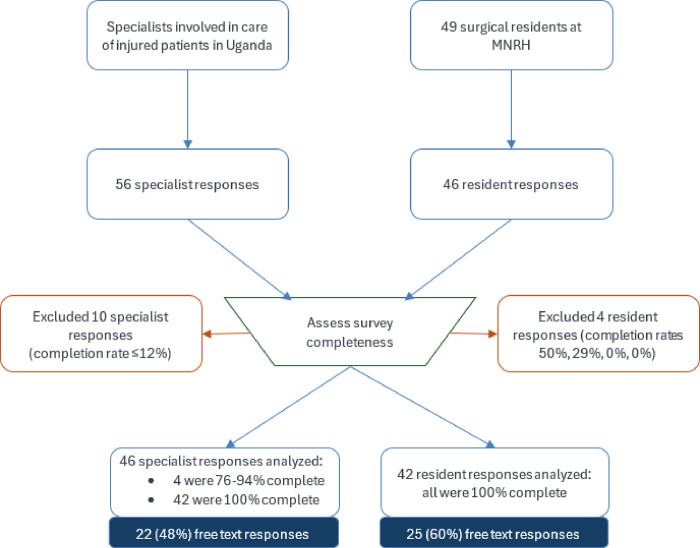
Flow diagram: selection of survey responses for analysis

After excluding 14 survey responses that were < 75% complete (Fig. 1), we analyzed responses from 46 consultants and 42 residents. Most were 31–40 years old and male (Table 1). As specified in sampling criteria, there was at least one consultant from each of 13 clinical disciplines involved in the care of injured patients. Among the general surgery residents training at MNRH, the response rate was 76% (target was 80%).

### Perceptions of Current State of Patient Care and Trauma Training

Most consultants strongly agreed (67%) or agreed (22%) that Uganda should have a surgeon specialized in the management of injured patients. Eighty-three percent of residents indicated they would consider a TFP and a career in trauma surgery; 59% would be interested in periodically working as a surgical critical care doctor (in an intensive care unit).

Most (67%) consultants and 54% of the 24 residents who had completed the trauma rotation reported being dissatisfied or very dissatisfied with the current state of care for injured patients in Uganda. The perceived challenges in providing quality care comprised human and material resource constraints, inadequate trauma systems (including prehospital care and trauma consultants), and inadequate in-hospital acute care (Table 2). Notably, 46% of residents who completed their trauma rotation only felt “somewhat prepared” to manage complex trauma patients; another 46% felt “adequately prepared,” and only 8% felt “very prepared.”

### Barriers and Motivating Factors in Establishing a TFP

When asked about the most likely barriers to surgeons pursuing a career in trauma (with or without a fellowship), 86% of consultants cited the lack of proper trauma systems and 75% cited the lack of a training program in Uganda. Only 25% were concerned about a lack of interest among trainees; 11% felt that trainees may be discouraged by the relatively high proportion of non-operative management.

Resident responses assuaged some of these concerns. Eighty-three percent of residents indicated that they would consider a TFP, a majority stating that “it would allow me to better take care of injured patients.” A significant minority were concerned this training would not be recognized by the government (31%) or that there is no accrediting body for trauma surgeons in Uganda (26%). Only 10% were worried about this being the first such fellowship in the region.

Consultants similarly felt the high burden of injured patients would be a motivating or facilitating factor in the establishment of a TFP (Table 3). Perceived barriers included the lack of trauma infrastructure, absence of a TFP curriculum, and challenging employment prospects after training.

### TFP Curriculum and Structure

To assist with curricular planning, consultants were asked to rank the procedures and skills from their clinical practice that every Ugandan trauma consultant should be able to perform (Table 4). When asked specifically about pediatric trauma, 98% of consultants stated that a TFP graduate should be familiar with the treatment of injured children.

Aside from dedicated trauma training, 91% of consultants and 76% of residents recommended the TFP include at least several short rotations on a critical care unit; 66% of both consultants and residents recommended it include emergency general surgery (EGS) training.

Regarding TFP logistics, 78% of consultants and 74% of residents preferred a program with exposure to both local and international settings. If a TFP were established in Uganda, 93% of consultants would be willing to host fellows from other countries. Those opposed cited concerns that Ugandan training sites would not have sufficient “equipment and order” to exemplify good trauma management, or that Uganda should prioritize its own “critical shortage of human resources for health” prior to supporting foreign trainees. There was no clear preference on the ideal duration of a TFP: a small plurality of consultants (41%) and residents (43%) preferred a 24-month (as opposed to 18- or 12-month) duration.

Finally, when asked about anticipated effects on existing training programs and clinical productivity, most consultants felt that a TFP would have a net positive influence on patient outcomes (93%), learning (including for non-TFP trainees, 77%), and clinical efficiency (68%). A minority were concerned that graduates of TFPs would add redundant physicians to the team (11%) or take learning opportunities away from other trainees (7%).

### Free Text Analysis

A total of 22 consultants (48%) and 25 residents (60%) provided optional free-text responses. Inductive and semantic coding revealed nearly all responses could be grouped into one of three themes: 1) the *need* for a TFP (55% consultants, 80% residents); 2) anticipated *potential challenges* during the implementation of a TFP in Uganda (36% consultants, 24% residents); 3) *suggestions* for successful implementation (41% consultants, 60% residents). Further analysis with a deductive approach with greater emphasis on latent content allowed for re-coding of these inductive themes into the themes from the multiple-choice survey: perceptions of trauma care and training, barriers and motivating factors affecting potential TFP development, and recommendations for TFP structure and educational objectives (Table 5). The findings are reported following those themes.

Regarding perceptions of trauma care and training, both consultants and residents were emphatically supportive of a potential TFP, stating that it is “long overdue” and has the potential to dramatically improve patient care. Consultants recognized the inadequacy of the current system of care for the injured patient, in which each specialist focuses on their “anatomical area of their interest,” and no consultant is responsible for the comprehensive care of the patient. Further, they underscored the need for effective prioritization of interventions for patients with complex or multisystem injuries.

Regarding barriers and motivating factors, awareness of the urgent need for trauma system strengthening emerged as a major motivating factor. Physicians also noted that “political will” and infrastructural support will be essential to maximize a TFP’s potential to improve outcomes for injured patients. They called for “access to theatre space as well as other diagnostic modalities,” with multiple residents emphasizing the need for “up-to-standard emergency units,” with “a proper and comfortable place to receive patients,” “basic instruments for diagnosis,” and “accessible and affordable” emergency equipment.

Regarding TFP structure, physicians indicated that trauma-trained consultants should learn to manage “patients efficiently enough to get them to their [definitive] specialist alive,” and “know when to call the specialist and not struggle alone.” Additional comments indicated that – to improve nationwide distribution of surgical workforce and secure buy-in from the Ministry of Health – trauma consultants “should be posted in regional referral hospitals.”

## Discussion

This is the first survey of clinicians from a low-income country regarding their perceptions of a potential TFP. It was motivated by local clinicians’ own interest in leveraging international partnerships to develop a TFP and by evidence suggesting that such a program may reduce injury-related mortality. We sought to assess whether this interest is shared among the larger community of Ugandan physicians involved in trauma care. Our survey results (1) indicate that Ugandan clinicians broadly support the establishment of a local TFP, (2) reveal perceived barriers and motivating factors in the TFP establishment process, and (3) offer suggestions for a preferred TFP curricular structure.

The chief finding from this survey is the nearly unanimous support among Ugandan physicians for a locally run TFP. Both the consultants who would potentially serve as TFP faculty and the residents who would be trainees of the TFP view it as an essential and long-overdue step toward improving care for injured patients in Uganda. These results are evidence of the contextual relevance of a TFP and buy-in from local clinicians – two factors that are vital to the success of any clinical training program [19, 22]. Furthermore, residents’ demonstrated interest in a potential career in trauma could facilitate recruitment of trainees into the proposed fellowship.

Our second major finding comprises the perceived barriers and facilitators in TFP development. According to consultants and residents, the main facilitators of TFP development are the high burden of injured patients and the belief that a TFP could substantially reduce the morbidity and mortality from injuries in the country. These perceptions align with the evidence. Systematic reviews indicate that certified, in-hospital, trauma training courses can reduce injury-related mortality in LMICs [19]. Given that TFPs do not yet exist in the COSECSA region, the only evaluation of TFP implementation in a comparable setting comes from Pakistan, a country that implemented a trauma quality improvement package, including the establishment of a two-year clinical fellowship and a trauma team with a dedicated leader [23]. A pre-post analysis of this package demonstrated sustained reductions in both mortality and operative complication rates [23]. In our survey, alongside overwhelming enthusiasm about a potential TFP, physicians are concerned about inadequate prehospital care, material resources, and critical infrastructure – known gaps in the trauma care pathway in Uganda [24]. Moreover, insufficient public financing forces hospitals like MNRH to request upfront payment from patients and families prior to providing care, delaying care and worsening health disparities. Together, these issues reflect the fact that a TFP is just one component of a broader set of interventions necessary to improve trauma care. Nevertheless, a thoughtful implementation of a TFP at MNRH could include organizational changes whose benefits extend beyond workforce strengthening [19, 25]. For example, establishing a dedicated trauma service [26], staffing a full-time trauma surgeon [27], implementing standardized trauma protocols [28], and increasing supervision of junior trainees [29] can all improve outcomes for injured patients.

Third, this survey reveals concerns about the availability of post-fellowship employment opportunities – a critical logistical consideration in TFP implementation. Although this has not been described in the literature, the authors’ anecdotal experience indicates the Ugandan government does not reliably adjust surgeons’ pay scales to reflect subspecialty expertise. However, the pathway toward accreditation of trauma as a surgical subspecialty could be accelerated based on precedent from other surgical subspecialties, including urology and pediatric surgery.

Finally, this survey gathered respondents’ preferences on how a TFP should be structured. As the field of trauma evolves toward non-operative management and inclusion of EGS, even settings with long-running TFPs are debating the optimal proportion of EGS and critical care training as part of a TFP[17]. This was addressed with a dedicated question in our survey: most respondents felt that a trauma fellowship should include a combination of trauma surgery, surgical critical care, and EGS, mirroring many U.S.-based TFPs, as well as the TFP organized by the West African College of Surgeons (WACS) [30]. Most of our survey respondents would also prefer a TFP with both local and international components; similarly, the WACS TFP curriculum requires two of the 12 months of fellowship to be completed in an established Level I trauma center, either in North America, Europe, or South Africa. While there was no clear consensus on the preferred duration of a Ugandan TFP, a plurality of respondents preferred a two-year fellowship. Collectively, these responses should be used to guide TFP curriculum development in Uganda.

As the first subspecialty trauma training program for the COSECSA region, a Ugandan TFP could capitalize on momentum toward establishing subspecialty surgical training to fill a critical gap in the regional trauma training landscape. To date, regional training programs have focused on the prehospital setting, front-line clinicians, and surgical residents – not on developing trauma and critical care subspecialists. Regarding prehospital care, a training course for Ugandan lay first-responders demonstrated that thoughtful piloting and application of WHO educational frameworks can result in knowledge retention and confidence in skill application, with favorable cost-effectiveness [8]. To improve primary- and secondary-survey proficiency among front-line healthcare workers, the primary trauma care (PTC) course was developed in 1997 [30] and implemented in 2012 through collaboration between international universities and COSECSA, successfully garnering financial support from health ministries of Rwanda, Kenya, and Uganda [13, 31]. An observational study showed improvement in knowledge, confidence and skill, as well as mortality reduction [32]. In 2007, the general surgeons at MNRH partnered with international collaborators to design the Kampala Advanced Trauma Course (KATC) for surgical interns. The KATC has since been disseminated to multiple hospitals throughout the country, underscoring the importance of the train-the-trainer model and challenges related to equipment availability, a concern also identified in our survey [33]. The two published reports of courses adapted for Ugandan surgical trainees are limited by sample size but suggest that a trauma operative skills course may improve knowledge (based on pre- and post-course tests) and comfort with intraoperative decision-making [34, 35]. All these courses have been favorably reviewed by their participants, indicating an eagerness for training opportunities that was replicated in our survey results.

This study has several limitations related to representativeness and generalizability of our results. First, quota sampling of consultants, while it provides perspectives from a broad range of clinical disciplines, does not ensure that responses represent the views of the overall population of specialists who would be involved in a TFP. Since the survey was distributed using professional society networks, it is not possible to know the overall response rate. Second, with a 76% response rate among all residents and only one respondent from the second-year cohort, we fell short of our target of 80%. This difference is small, and, given the consistency in the respondents’ attitudes towards a TFP, unlikely to change our main findings. Finally, we acknowledge that this study, by design, is highly context-specific; the attitudes expressed by respondents may not be generalizable to other settings. Although this survey focuses on one low-income country in Africa, we believe that its findings - including physicians’ acute awareness of the need for trauma systems strengthening, their eagerness for training opportunities, and the importance of external factors on TFP success - are broadly applicable to other settings with limited resources to address high burdens of injury.

Future work will involve engaging additional stakeholders in the surgical training process to gather input and secure support for implementing the proposed TFP. Key stakeholders not represented in this survey include MNRH leadership, faculty from international Level I trauma centers, Ugandan health ministers, and COSECSA leadership. We will incorporate feedback from these groups, integrate insights from our survey results, and draw on programmatic frameworks of established TFPs to develop a comprehensive plan for accreditation and implementation.

## Conclusion

This cross-sectional survey addressed a prerequisite question for the implementation of any clinical training program: is there support from the clinicians who would serve as faculty and trainees of a proposed TFP? The answer is unambiguously affirmative, and free-text responses underscore the urgent desire for subspecialty training for physicians to more confidently and safely address the heavy burden of injury in Uganda. These results indicate that MNRH may be a favorable site for the first TFP for Uganda and provide actionable recommendations for curriculum development.

## Figures and Tables

**Table 1:** Baseline demographics and clinical experience of survey respondents

	Residents (N = 42)		Consultants (N = 46)	
Demographics				
Age Distribution				
20–30 years	6 (14%)		0%	
31–40 years	33 (79%)		30 (65%)	
41–50 years	3 (7%)		8 (17%)	
51 + years	0 (0%)		8 (17%)	
Gender				
Female	8 (20%)		9 (20%)	
Male	33 (80%)		35 (80%)	
Clinical Background				
	**Completed trauma rotation**		**Proportion of injured patients in current clinical practice**	
	No	18 (43%)		
	Yes	24 (57%)	0%	3 (3%)
	**Year of residency**		1–25%	22 (48%)
	1	16 (42%)	26–50%	11 (24%)
	2	1 (3%)	51–75%	7 (15%)
	3	21 (55%)	76–100%	3 (3%)
			**Clinical Specialty**	
			General surgery	14 (30%)
			OB/GYN	8 (17%)
			Urology	7 (15%)
			Cardiothoracic surgery	4 (8%)
			Neurosurgery	3 (6%)
			ENT surgery	2 (4%)
			Orthopedic surgery	2 (4%)
			Critical care / anesthesia	1 (2%)
			Emergency medicine	1 (2%)
			Ophthalmology	1 (2%)
			OMF surgery	1 (2%)
			Plastic and reconstructive surgery	1 (2%)
			Upper GI and HPB surgery	1 (2%)

Results presented as number (percent total); column totals may not add up to sample size if a question was skipped. OB/GYN: obstetrics and gynecology; ENT: ear, nose, and throat; OMF: oral and maxillofacial; GI: gastrointestinal; HPB: hepatopancreaticobiliary.

**Table 2:** Perceived challenges in caring for injured patients in Uganda

Challenge	Residents	Consultants
Insufficient/inadequate material resources	36 (86%)	41 (89%)
Delayed/late presentation to hospital	31 (74%)	38 (83%)
Inappropriate/inadequate resuscitation	30 (71%)	36 (78%)
Insufficient/inadequate human resources	29 (69%)	37 (80%)
Inadequate inpatient care despite good ED resuscitation	25 (60%)	17 (37%)
No trained consultants in trauma	22 (52%)	19 (41%)

**Table 3:** Barriers and motivating factors for establishing a TFP in Uganda, according to consultants

Barriers	Percent of Respondents	Motivating Factors	Percent of Respondents
Lack of trauma infrastructure (supplies, staff, theatre time/space)	36 (82%)	High burden of injured patients	31 (70%)
Lack of TFP curriculum	31 (70%)	Established accreditation pathway in other specialties (urology, pediatric surgery)	29 (66%)
Poor employment prospects after training	29 (66%)	Motivated faculty	27 (61%)
Lack of funding	27 (61%)	Motivated residents	22 (50%)
No available faculty to train the fellows	26 (59%)	Potential for political/ministry support	21 (48%)
Lack of accreditation (certifying body) at present	23 (52%)		

TFP: trauma fellowship program

**Table 4:** Procedures and skills in which a Ugandan trauma surgeon should be proficient, as identified by consultants, listed in order of ranked importance

Clinical Specialty	Skills and Procedures
General surgery	Splenectomy, complex wound debridement, small intestine resection and anastomosis (± adhesiolysis)
OB/GYN	Cesarean section, manual / vacuum-assisted evacuation, repair of perineal tear
Urology	Bladder repair, nephrostomy tube placement, ureteral repair
Cardiothoracic surgery	Tube thoracostomy, thoracotomy, vascular repair
Neurosurgery	Decompressive craniotomy (Burr hole), craniectomy
ENT surgery	Epistaxis management, neck exploration with possible vascular or tracheal repair
Orthopedic surgery	Fasciotomy, external fixation, limb amputation
Critical care / anesthesia	Advanced airway management (e.g., intubation), hypovolemic shock, sedation
Emergency medicine	Tube thoracostomy, wound closure, initial fracture management
Ophthalmology	Management of chemical eye injuries, lateral canthotomy
OMF surgery	Repair of facial fractures
Plastic and reconstructive surgery	Resuscitation of severely burned patients, burn debridement / escharotomy, skin grafting
Upper GI and HPB surgery	Splenectomy, damage-control surgery, partial hepatectomy

OB/GYN: obstetrics and gynecology; ENT: ear, nose, and throat; OMF: oral and maxillofacial; GI: gastrointestinal; HPB: hepatopancreaticobiliary

**Table 5:** Representative quotes from thematic analysis of free-text responses from 22 consultants and 25 residents, grouped by theme

State of Patient Care and Trauma Training	Motivating Factors	Potential Barriers	Structuring a TFP
Consultants currently caring for trauma patients “are not really interested in the trauma patient unless it involves an anatomical area of their interest” (consultant)	“Patient survival would increase exponentially” (consultant)	“Trainees worry that the [trauma] specialty [would] not offer opportunities for lucrative private practice,” while “specialists working in public hospitals” would only receive “poor pay by government” (consultant)	Trainees entering TFP should “come from internship [and] one year [as] a medical officer; otherwise […] it’s overlapping with surgical training.” (consultant)
“Uganda is overwhelmed with trauma, [with an] urgent need for better [triage] and emergency systems” (consultant)	“This will be the best thing in as far as trauma care in Uganda is concerned” (consultant)	“Political will” is critical in supporting “pay structure, jobs, [andftraining, or else external funding” will be necessary (faculty)	TFP “should be open to all [surgical] disciplines, not only general surgery” (consultant)
“All regional [referral centers] in Uganda are wanting in terms of trauma management” (resident)	TFP “is long overdue” and “should start immediately” (consultant)	“Up to standard emergency units should be built […] in Uganda” (resident)	“Career path,” including “employment after training,” “should be clear” with recognition of the “specialty structure within the Uganda government” (consultant)
“Advocacy for starting the fellowship at Mulago and other universities in Uganda should be a priority.” (resident)	“Should have had this specialized training start more than 20 years ago!” (resident)	“Provide basic instruments for diagnosis” (resident)	“To get buy-in from MoH,” it should be emphasized “that [trauma consultants] should be posted in regional referral hospitals” (consultant)
		Setup “at Mulago may not be favourable for the training” (consultant)	Trauma “fellowship should be accredited here in Uganda” (resident)

TFP: trauma fellowship program, MoH: Ministry of Health

## Data Availability

All data generated or analyzed during this study are included in this published article and its supplementary information files “Additional file 1 (survey forms)” and “Additional file 2 (survey responses)”.

## Abbreviations

COSECSA: College of Surgeons of East, Central and Southern Africa
CROSS: Consensus-Based Checklist for Reporting of Survey Studies
EGS: emergency general surgery
KATC: Kampala Advanced Trauma Course
LMICs: low- and middle-income countries
MNRH: Mulago National Referral Hospital
TFP: trauma subspecialty (fellowship) training program
WACS: West African College of Surgeons
